# Cyclic stretch augments human rhinovirus induced inflammatory responses in airway epithelial cells

**DOI:** 10.1186/1710-1492-10-S1-A71

**Published:** 2014-03-03

**Authors:** Sergei Nikitenko, Sami Shariff, Jason Arnason, Chris Shelfoon, Cora Kooi, David Proud, Richard Leigh

**Affiliations:** 1 Snyder Institute for Chronic Diseases, University of Calgary, Calgary, Alberta, T2N 4N1, Canada

## Background

Structural cells of the airways are subject to normal mechanical stretch during respiration [1]. Mechanical stretch acts as a mechanism of cell activation, and mechanotransduction pathways have been shown to activate pro-inflammatory genes [2]. Human rhinovirus (HRV) infections are a major cause of asthma exacerbations, and mechanical forces are likely to be more pronounced during asthma exacerbations [3]. Moreover, smoking is associated with worse clinical outcomes in asthma. Previous studies have shown that HRV infection, cigarette smoke extract (CSE), or mechanical stretch each induce CXCL8 production in bronchial epithelial cells [4-6]. In this study, we sought to determine whether mechanical stretch interacts with HRV infection and CSE to further upregulate airway inflammation.

## Methods

Studies were performed using primary human bronchial epithelial cells (HBEC), the human bronchial epithelial cell line BEAS-2B. Cells were treated with CS (FlexCell FX-4000), HRV-16 or with a combination of CS+HRV-16. Protein and mRNA levels were measured using ELISA (R&D Systems) and real-time RT-PCR (Applied Biosystems).

## Results

Mechanical stretch and HRV infection each significantly increased CXCL8 release in BEAS-2B and HBE cells compared to static controls (p<0.001). When studied in combination, there was a significant synergistic increase in CXCL8 in BEAS-2B (p<0.001). Ultraviolet (UV) inactivation of HRV attenuated this increase in CXCL8 release. Mechanical stretch and HRV infection each increased CXCL8 mRNA levels compared to static controls, with the combination resulting in further enhanced induction (p<0.01). CSE alone did not significantly increase CXCL8 production in BEAS-2B. However, the combination of CSE+HRV significantly enhanced CXCL8 release compared to medium control (p<0.001) and HRV alone (p<0.05), and this increase was significantly further augmented by mechanical stretch (p<0.001).

## Conclusions

Mechanical stretching of BEAS-2B and HBE cells increased HRV-induced CXCL8 mRNA and protein levels, confirming that this effect is, at least in part, regulated at the transcriptional level. Moreover, the combination of mechanical stretch and HRV infection, as might occur during asthma exacerbations, resulted in a synergistic enhancement of CXCL8 expression, compared to either stimulus alone. CSE further enhanced HRV-induced and stretch-induced CXCL8 release, and the combination of all three variables potentiated this effect. Since levels of CXCL8 have been linked to increased airway neutrophils and symptom severity, mechanical stretch, if it also enhances CXCL8 production induced during HRV infections *in vivo*, may contribute to the pathogenesis of airway inflammation in HRV-induced asthma exacerbations.
